# Incidence and Clinical Predictors of Ocular Candidiasis in Patients with *Candida* Fungemia

**DOI:** 10.1155/2014/650235

**Published:** 2014-11-17

**Authors:** Ayesha Khalid, Lisa A. Clough, R. C. Andrew Symons, Jonathan D. Mahnken, Lei Dong, Albert J. Eid

**Affiliations:** ^1^Division of Infectious Diseases, University of Kansas Medical Center, 2067 Delp, 3901 Rainbow Boulevard, Kansas City, KS 66160, USA; ^2^Department of Ophthalmology, Royal Melbourne Hospital, Parkville, VIC 3050, Australia; ^3^Department of Biostatistics, University of Kansas Medical Center, Kansas City, KS 66160, USA

## Abstract

*Purpose*. The aim of this study is to determine the incidence and the predictors of ocular candidiasis among patient with* Candida* fungemia.* Methods*. We retrospectively reviewed the charts of all patients diagnosed with candidemia at the University of Kansas Medical Center during February 2000–March 2010. Data regarding patients' demographics, clinical characteristics, laboratory results, and ophthalmology examination findings were collected.* Results*. A total of 283 patients with candidemia were enrolled. The mean age (± standard deviation) was 55 ± 18 years; 66% were male. The most commonly isolated* Candida* species were* C. albicans* (54%),* C. parapsilosis* (20%),* C. glabrata* (13%), and* C. tropicalis* (8%). Only 144 (51%) patients were evaluated by ophthalmology; however, the proportion of patients who were formally evaluated by an ophthalmologist increased during the study period (9%in 2000 up to 73%in 2010; *P* < 0.0001). Evidence of ocular candidiasis was present in 18 (12.5%) patients. Visual symptoms were reported by 5 of 18 (28%) patients. In multivariable analysis, no predictors of ocular candidiasis were identified.* Conclusions*. The incidence of ocular candidiasis among patients with fungemia remains elevated. Most patients are asymptomatic and therefore all patients with candidemia should undergo fundoscopic examination to rule out ocular involvement.

## 1. Introduction

The rate of hospital-acquired bloodstream infections due to* Candida* species increased significantly over the past few decades [1–3]. In 2002,* Candida* accounted for 12% of all bloodstream infections acquired in US hospitals, with an estimated incidence of 4.6 per 10,000 admissions [3].* Candida* bloodstream infections are associated with increased morbidity and mortality [4]. Therefore, appropriate identification and treatment of these infections as well as their associated complications is a high priority.

One major complication of* Candida* bloodstream infection is the hematogenous seeding of the eye, leading to chorioretinitis or endophthalmitis (chorioretinitis associated with vitritis). If untreated, endophthalmitis can result in retinal necrosis and detachment with devastating visual consequences. Early identification and adequate treatment of this complication is critical to preserving vision. Underscoring the importance of identifying ocular candidiasis, the guidelines of the Infectious Diseases Society of America (IDSA) for the management of candidiasis recommend that all patients with candidemia should undergo ophthalmological examination [5]. Furthermore, the panel suggests that screening fundoscopy should be used as a performance measure in the management of candidiasis. Data are lacking, however, regarding the compliance of physicians with these recommendations.

The data regarding the incidence of ocular candidiasis is somewhat conflicting. Furthermore, it is not clear whether the observations that were made more than twenty years ago are still valid. Recent data suggest that although candidemia is becoming more common, the incidence of ocular candidiasis appears to be decreasing. Studies published prior to 1990 reported incidence rates ranging from 28 to 37% [6, 7], while most studies published after 1990 reported lower incidence rates (<2–11.6%) [8–14]; however, discrepancies between studies persist [15–17]. The reason for the apparent decline in* Candida* ocular infection has not been well established. Some authors suggest that the evolving epidemiology of* Candida* species may account for this change. Recent studies demonstrate that among patients with candidemia,* Candida* non-*albicans* species now account for a greater proportion of isolates (54.5%–60.3%) than* Candida albicans* (39.7%–45.6%) [4, 14]. However, the prevalence of* Candida* species is likely to vary considerably among institutions and in different geographical locations.* C. albicans* may confer greater risk for ocular disease over other* Candida* species [8, 13–15], hence the declining rate of ocular candidiasis. Another possible explanation is the early initiation of systemic antifungal therapy in patients with candidemia, which has become a common practice. Whether screening all patients for ocular involvement in an era when* Candida* non-*albicans* species are more prevalent and when patients are more likely to receive therapy in a timely manner is cost-effective remains to be determined.

In this single-center* retrospective* study, we determined the proportion of patients with candidemia who underwent formal ophthalmological evaluation during the 10-year study period. We also measured the incidence of ocular candidiasis among our patients and attempted to identify clinical predictors of ocular candidiasis.

## 2. Patients and Methods

### 2.1. Study Design

Adult patients (>18 years) who were diagnosed with* Candida* fungemia during February 2000–March 2010 at the University of Kansas Medical Center were identified using the microbiology laboratory database and electronic medical records. The charts were retrospectively reviewed and patients' demographics, clinical characteristics, and microbiology data were collected using a standardized case report form. Furthermore, we abstracted data regarding patients' comorbidities such as diabetes, need for hemodialysis, and malignancies, immunosuppression (i.e., transplantation, steroid use, HIV infection/AIDS, and neutropenia), prior antibiotic therapy, parenteral nutrition, and severity of illness (i.e., Apache II score, need for care in the intensive care unit, and mechanical ventilation). Microbiology data regarding the number of blood cultures growing* Candida* species, duration of candidemia, and the identity of* Candida* species isolated from the blood were collected. The proportion of patients with candidemia who underwent formal ophthalmological evaluation was determined throughout the study period. The timing of the ophthalmological evaluation and the findings of fundoscopic examination were recorded. The outcome of interest was ocular involvement due to* Candida* infection (i.e., chorioretinitis and vitritis). Patients with negative initial fundoscopic examination underwent repeat examination every week as long as they remained hospitalized. One of the authors (AS) reviewed the ophthalmological findings and determined if the patients had sufficient evidence of ocular candidiasis.

### 2.2. Definitions

Ocular candidiasis was diagnosed based on the findings of the fundoscopic examination and it was defined as the presence of chorioretinitis with or without vitritis. Chorioretinitis was defined as the presence of white chorioretinal infiltrates in the absence of vitreal cells or “fluff balls.” On the other hand, vitritis was defined as the presence of vitreal cells or “fluff balls.” Endophthalmitis meant the presence of both chorioretinitis and vitritis. The duration of candidemia was defined as the number of days that separates the first positive and the first negative blood cultures; the timing of repeat blood cultures was determined by the treating physicians.

### 2.3. Statistics

Descriptive statistics were used to characterize the study population and determine the incidence of chorioretinitis and or endophthalmitis among patients with candidemia. In order to determine the risk factors associated with chorioretinitis or endophthalmitis, bivariate analyses were conducted. For categorical measures, Pearson's chi-square tests or Fisher's exact tests were used. For continuous measures, the distributions were compared between those with and those without ocular candidiasis using the Wilcoxon rank sum test. The Cochrane-Armitage test for trend was used to evaluate changes in proportions over time. Finally, an unconditional logistic regression model with ocular candidiasis as the response measure was built using stepwise selection of the explanatory variables used for the bivariate analyses. The model entry criterion was a score test *P* value less than 0.2, and the criterion to stay in the model was a Wald test *P* value less than 0.15. Unadjusted odds ratios and 95% Wald confidence intervals were also generated for these candidate explanatory measures with ocular candidiasis as the response measure. All data management and data analysis were performed using SAS version 9.2 (2002–2008, SAS Institute Inc., Cary, NC).

## 3. Results

### 3.1. Patient Population

During the study period starting on February 1, 2000, and ending on March 31, 2010, a total of 311 adult patients were diagnosed with* Candida* fungemia at the University of Kansas Medical Center. Only 283 patients were included in the study due to missing data regarding 28 patients. The mean age (±standard deviation [SD]) of the patients was 55 ± 18 years and 66% were male. Treatment in the intensive care unit (ICU) was required for 169 (60%) patients. The mean (±SD) duration of stay in the ICU was 11.7 ± 20.8 days. Mechanical ventilation was required in 142 (50%) patients and 121 (43%) patients were receiving total parenteral nutrition (TPN). Sixteen patients (6%) had a history of IV drug use. The characteristics of the patient population are presented in Table 1.

### 3.2. Characteristics of* Candida* Fungemia

**Figure 1:**
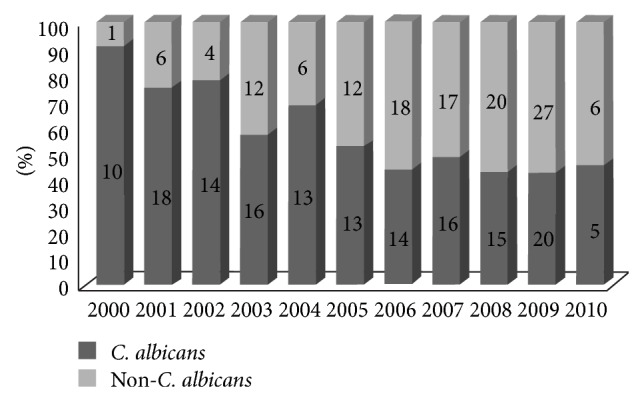
Proportion of* C. albicans* versus non-*C. albicans* species during the study period.

Among the patients enrolled in the study (*n* = 283), the mean (±SD) number of blood cultures growing* Candida *species was 1.9 ± 1.4 and the mean (±SD) duration of fungemia was 5.8 ± 7.6 days. The predominant* Candida* species was* C. albicans* 54%, followed by* C. parapsilosis* (20%),* C. glabrata* (13%),* C. tropicalis* (8%),* C. krusei* (1%),* C. dubliniensis* (1%), and* C. lusitaniae* (1%). The prevalence of* C. albicans* decreased significantly during the study period (91% in 2000 down to 43% in 2009 and 45% in 2010; *P* < 0.0001), Figure 1. The mean (±SD) time to initiation of antifungal therapy among those who were not already on treatment (*n* = 259) was 1.75 ± 1.5 days.

### 3.3. Fundoscopic Examination of Patients with Candidemia

**Figure 2:**
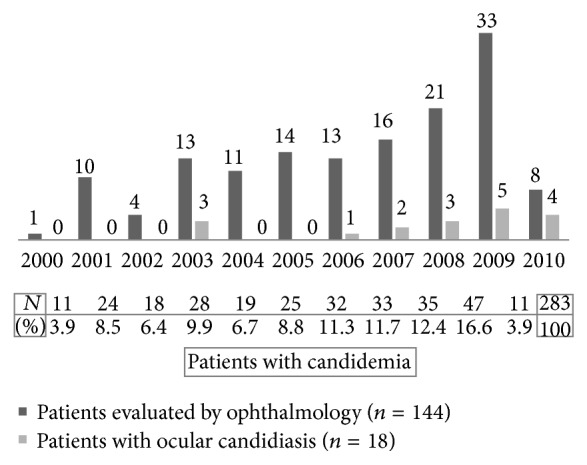
Candidemia and ocular candidiasis during the study period.

During the study period, 144 out of 283 patients (51%) underwent a fundoscopic examination by an ophthalmologist, at least on one occasion, at the request of the treating physician. The mean time (±SD) from the collection of positive blood culture to fundoscopic examination was 5.8 (±5.8) days. Repeat eye exam was performed on 18 of 144 patients only (13 of those patients had already been diagnosed with ocular candidiasis; 5 patients had negative first fundoscopic examination). None of the patients with ocular candidiasis was diagnosed at the time of repeat examination. In our study, patients with candidemia were more likely to receive formal ophthalmological evaluation during the later part of study period (rate increased from 9% during 2000 to 73% in 2010; *P* < 0.0001), Figure 2.

### 3.4. Incidence of Ocular Candidiasis

Among 144 patients with* Candida* fungemia who underwent ophthalmological evaluation, 18 (12.5%) were diagnosed with ocular candidiasis. All 18 patients had evidence of chorioretinitis; only 2 of them had vitritis as well (i.e., endophthalmitis). Eleven patients had involvement of a single eye (right eye in 6 patients; left eye in 5 patients) while 7 patients had simultaneous involvement of both eyes. Only 5 out of 18 (33%) patients reported visual symptoms prior to diagnosis. The proportion of patients found to have ocular candidiasis varied over the years (Figure 2) and was higher towards the end of the study period (*P* = 0.0016).

### 3.5. Predictors of Ocular Candidiasis

The analysis of factors associated with ocular candidiasis was limited to patients who underwent fundoscopic examination by an ophthalmologist (*n* = 144). Among those, 18 (12.5%) were diagnosed with ocular candidiasis. Bivariate analyses were initially performed, the results of which are presented in Table 2. None of the studied predictors were significantly associated with ocular candidiasis; however, the use of steroids (*P* = 0.042) and IV drug use (*P* = 0.053) showed a trend towards association. The multivariable model showed no significant association of steroid use (OR 0.316 [95% confidence limits 0.086–1.164]; *P* = 0.13) or IV drug use (OR 3.138, [95% confidence limits 0.692–14.241]; *P* = 0.06) with ocular candidiasis; Table 3.

## 4. Discussion

This study shows that the current incidence of ocular candidiasis among patients with* Candida* fungemia remains elevated (12.5%) despite prompt initiation of antifungal therapy. The compliance of physicians with the IDSA's recommendation to obtain formal ophthalmological evaluation of patients with candidemia seems to have improved at our institution since 2004; however, further improvement is needed. We did not identify predictors of ocular candidiasis but IV drug use showed a trend towards an association.

The overall incidence of ocular candidiasis in our patient population (12.5%) is comparable to the incidence observed in similar studies with sizeable number of patients [8, 11, 14]. Higher and lower rates have been reported in other studies, either enrolling a limited number of patients [6, 7, 16, 17] or using a broader definition of systemic* Candida* infection (i.e., not limited to* Candida* fungemia) [12, 18]. Disparities among studies could also be attributed to technical differences such as personnel performing the fundoscopic examination (i.e., ophthalmologist versus other physicians) and performing pupils' dilation or not. In our patient population, ocular candidiasis was more likely to be diagnosed during 2006–2010 than earlier. This finding is likely to be explained by the fact that more cases of candidemia were seen at our institution during the more recent years and higher proportion of patients with candidemia received ophthalmological evaluation during this period.

The timing of fundoscopic examination might affect the reported incidence of ocular candidiasis. Early examination can lead to negative results in case follow-up examination is not performed. In a recent study by Nagao et al. 11 of 54 patients were diagnosed with ocular candidiasis more than 8 days after positive blood culture [15]. Oude Lashof et al. reported that 11 of 60 patients with ocular candidiasis (3% of patients with candidemia) were diagnosed at the time of follow-up examination [14]. In another study, 3 out of 8 patients found to have chorioretinitis were diagnosed 1 or 2 weeks following the initial examination despite antifungal therapy with Amphotericin B and or Fluconazole [16]. Similarly, two other studies have reported that 1 of 9 (11%) and 1 of 11 (9%) cases of ocular candidiasis were diagnosed at 1-week follow-up [6, 7]. That being said, the timing of the ophthalmological evaluation does not account for all the differences in terms of incidence of ocular candidiasis. Even when the initial examination was performed within 72 hours of positive blood culture, the incidence varied significantly among studies [8, 16]. In our study, the average time to fundoscopic examination after obtaining positive blood culture was 5.82 days. Only 12.5% of our patients had their eyes examined within 48 hours of the collection of positive blood culture. Even though weekly evaluation of patients with negative ocular findings was sought, the majority of our patients were not hospitalized long enough to undergo repeat fundoscopic examination, which was performed on 18 patients only (5 of them had negative first fundoscopic examination). None of the patients with ocular candidiasis were diagnosed at the time of repeat examination. The late detection of ocular involvement is likely to dramatically affect the treatment because some patients might be receiving an antifungal agent with poor penetration into the eye tissue (e.g., echinocandins). In addition, the duration of treatment for ocular candidiasis should be extended beyond the standard two-week course for candidemia and is dictated by the improvement of the ocular lesions. Given the significant consequences and the lack of conclusive evidence, the need for repeat fundoscopic examination requires further investigation in large prospective studies allowing clear recommendations for physicians managing patients with* Candida* fungemia. In fact, the current IDSA guidelines do not recommend repeat fundoscopic examination if the initial examination is negative due to lack of evidence [5].

It is obvious from our data that many patients with* Candida* fungemia did not receive fundoscopic examination during the first few years of the study (2000–2004) at a time when the IDSA guidelines for the management of candidiasis did not exist. During those years 35% of our patients were evaluated by ophthalmology, while during the second half of the study period, 66% of our patients received fundoscopic examination. This finding highlights the importance of guidelines and how they do impact the clinical practice. Even though improvement occurred during the past decade, better compliance is needed in order to ensure that all patients with candidemia are receiving the standard of care. Since the incidence of ocular candidiasis remains elevated, the recommendation of the IDSA to screen all patients with* Candida* fungemia for ocular candidiasis should be emphasized, because it is likely to result in better patient care.

No risk factors for ocular candidiasis were identified in this study. Fungemia due to* C. albicans* was associated with ocular involvement in previous studies [8, 13–15]. In our study, a similar association was not found. In fact, the incidence of* C. albicans* fungemia decreased significantly during the study period (Figure 1) while the number of patients diagnosed with ocular candidiasis increased. Since most patients with* Candida* fungemia were not screened for ocular candidiasis in the first part of the study, our results could be biased. As a result, an association between* C. albicans* and ocular candidiasis cannot be excluded. Other factors previously associated with ocular candidiasis, such as multiple positive blood cultures, immunosuppression, and visual symptoms, did not predict the presence of ocular disease in our patients [8, 13].

The current study has certain limitations. Due to the retrospective nature, a number of patients had missing data regarding at least one of the studied variables (*n* = 28) and therefore were excluded from the main analysis of predictors of ocular candidiasis. As a result, the study lost power. In our patients, the blood cultures were repeated as often as determined by the treating physician and not on a daily basis; therefore, the duration of candidemia might not be accurate. Not all patients underwent ophthalmological evaluation, which could have introduced bias affecting the incidence of ocular candidiasis. Another limitation is that only a small number of patients with negative initial fundoscopic examination underwent repeat eye exam, which did not allow us to assess the contribution of follow-up fundoscopy to detect ocular candidiasis.

In conclusion, ocular candidiasis remains common among patients presenting with* Candida* fungemia. All patients presenting with candidemia should undergo screening fundoscopy and that should be used as a performance measure in the management of candidiasis. Clinical characteristics were not helpful to predict which patients are likely to have ocular involvement. Prospective studies using systematic and weekly fundoscopic examinations as well as serial blood cultures are needed in order to determine the exact incidence of ocular candidiasis and identify possible risk factors. Given that echinocandins, which are considered first line agents to treat candidemia, have no intraocular activity, the impact of their use on ocular candidiasis should be investigated.

## Figures and Tables

**Table 1:** Patients characteristics.

Characteristics	Overall study cohort	Patients with eye examination	Ocular candidiasis present
	*n* (%)	*n* (%)	*n* (%)
	(*n* = 283)	(*n* = 144)	(*n* = 18)
Age in years (mean ± SD)	55 ± 18	54 ± 18	55 ± 11
Male gender	188 (66)	98 (68)	12 (67)
History of DM	59 (21)^e^	36 (25)	4 (22)
Mechanical ventilation	142 (50)^e^	71 (49)	7 (39)
TPN	121 (43)	70 (49)	8 (44)
History of malignancy	93 (33)^e^	38 (26)	2 (11)
Transplant recipient	19 (7)^e^	10 (7)	1 (6)
Prolonged immunosuppression^a^	51 (18)	19 (13)	1 (6)
Receiving steroids^b^	103 (36)	55 (38)	3 (17)
Hemodialysis	64 (23)	32 (22)	2 (11)
Neutropenia (ANC < 500)	12 (4)^e^	5 (3)	1 (6)
Abdominal surgery	80 (28)^e^	45 (31)	6 (33)
IVDU	16 (6)	9 (6)	3 (17)
HIV infection	1 (0.35)^e^	0	0
Patients with burns	18 (6)	10 (7)	2 (11)
Use of vascular catheter^c^	258 (91)^e^	135 (94)	18 (100)
Duration of candidemia^d^ (mean ± SD)	5.8 (±7.6) (73 missing)^g^	5.9 (±8.2) (18 missing)^g^	3.9 (±2.3)^e^
Number of (+) BC (mean ± SD)	1.9 ± 1.4	2.0 ± 1.6	2.2 ± 2.2
Apache Score (mean ± SD)	14.4 ± 7.4 (6 missing)	13.1 ± 6.64	14 ± 7.6
Time from (+) BC to fundoscopy^d^	5.82 ± 5.81	5.82 ± 5.81	4.89 ± 3.71
Time from (+) BC to AFT^d^	0.68 ± 4.70 (37 missing)	0.69 ± 4.54	0.83 ± 2.23
ICU admission	169 (60)	86 (60)	9 (50)
Duration of stay in ICU^d^ (mean ± SD)	11.7 ± 20.8	15.8 ± 25.9	18.8 ± 16.6
Length of hospital stay^d^ (mean ± SD)	32.9 ± 35.2^f^	39.1 ± 36^e^	28.3 ± 17.6
Mortality	67 (24)^f^	18 (12.5)	2 (11)
Eye pain	1 (0.35)^e^	1 (0.7)	1 (5.6)
Blurred vision	12 (4)^e^	12 (8)	3 (17)
Floaters	7 (2)^e^	7 (5)	2 (11)
Other symptoms^h^	10 (4)^e^	9 (6)	3 (17)

*Note*. ^a^Patients receiving corticosteroids, immunosuppressive medications, or chemotherapy; ^b^dose >15 mg of Prednisone or its equivalent daily for at least 3 weeks; ^c^arterial or central venous catheters; ^d^in days; ^e^one patient with missing data; ^f ^two patients with missing data; ^g ^number includes patients with no repeat blood culture available; ^h^various types of light perception, photophobia, burning, or pressure; AFT: antifungal therapy; ANC: absolute neutrophil count; BC: blood culture; ICU: intensive care unit; IVDU: intravenous drug use; SD: standard deviation; TPN: total parenteral nutrition.

**Table 2:** Bivariate analysis of factors associated with ocular candidiasis.

Variables	No ocular candidiasis *N* = 126	Ocular candidiasis *N* = 18	*P* value
Age (in years)	54 ± 19	55 ± 11	0.96
Male gender, *n* (%)	86 (68)	12 (67)	0.89
Number of positive BC (mean ± SD)	2 ± 1.5	2.2 ± 2.2	0.88
Time from (+) BC to fundoscopy^a^	6.0 ± 6.0	4.9 ± 3.7	0.53
Time from (+) BC to AFT^a^	0.7 ± 4.8	0.8 ± 2.2	0.27
Total parenteral nutrition, *n* (%)	62 (49)	8 (44)	0.71
Medical care in the ICU, *n* (%)	77 (61)	9 (50)	0.37
HD during the same hospitalization, *n* (%)	30 (24)	2 (11)	0.23
Steroid use, *n* (%)	52 (41)	3 (17)	0.045
Intravenous drug use, *n* (%)	6 (4.8)	3 (17)	0.051
Chronic immunosuppression, *n* (%)	18 (14)	1 (6)	0.3
Apache score (mean ± SD)	13 ± 6.5	14 ± 7.6	0.81
*Candida albicans* versus *Candida *non-*albicans*, *n* (%)	61 (48)	11 (61)	0.32

*Note*. ^a^In days; AFT: antifungal therapy; BC: blood cultures; HD: hemodialysis; ICU: intensive care unit; SD: standard deviation.

**Table 3:** Odds ratio of factors associated with ocular candidiasis (*n* = 144).

Variables	OR	95% CL	Adjusted OR	95% CL
Age (in years)	1.002	(0.974, 1.03)	—	—
Male gender	1.075	(0.376, 3.07)	—	—
Number of positive BC	1.06	(0.788, 1.426)	—	—
Time from (+) BC to fundoscopy	0.956	(0.848, 1.079)	—	—
Time from (+) BC to AFT	1.009	(0.897, 1.135)	—	—
Total parenteral nutrition	0.826	(0.306, 2.229)	—	—
Medical care in the ICU	0.636	(0.236, 1.714)	—	—
HD during the same hospitalization	0.4	(0.087, 1.84)	—	—
Steroid use	0.285	(0.078, 1.033)	0.316	(0.086, 1.164)
Intravenous drug use	4	(0.905, 17.683)	3.138	(0.692, 14.241)
Chronic immunosuppression	0.353	(0.044, 2.818)	—	—
Apache score	1.023	(0.952, 1.099)	—	—
*Candida albicans* versus *Candida *non-*albicans *	1.674	(0.610, 4.597)	—	—

*Note*. AFT: antifungal therapy; BC: blood cultures; CL: confidence limits; HD: hemodialysis; ICU: intensive care unit; OR: odds ratio.
